# Redox/pH-Responsive 2-in-1 Chimeric Nanoparticles for the Co-Delivery of Doxorubicin and siRNA

**DOI:** 10.3390/polym13244362

**Published:** 2021-12-13

**Authors:** Hsi-Chin Wu, Wei-Ting Kuo

**Affiliations:** Department of Mechanical and Materials Engineering, Tatung University, Taipei 104, Taiwan; giftedkuo@gmail.com

**Keywords:** co-delivery, pH responsive, redox responsive, targeted delivery

## Abstract

The co-delivery of chemotherapy drugs and gene-suppressing small interfering RNA (siRNA) show promise for cancer therapy. The key to the clinical realization of this treatment model will be the development of a carrier system enabling the simultaneous delivery (“co-delivery” instead of combinatorial delivery) of chemotherapy and siRNA agents to cancer. In this study, a co-delivery system was developed from two individual components to form one integrated nanovehicle through a redox-sensitive thiol–disulfide bond for the synergistic delivery of chemotherapy and RNA silencing: doxorubicin (Dox)-loaded N,O-carboxymethyl chitosan (NOCC) complex with a thiolated hyaluronic acid (HA-SH) nanocarrier and dopamine (Dopa)-conjugated thiolated hyaluronic acid (SH-HA-Dopa)-coated calcium phosphate (CaP)-siRNA nanocarrier. The 2-in-1 chimeric nanoparticles (NPs) were structurally stable together in the storage environment and in the circulation. This smart system selectively releases Dox and siRNA into the cytosol. Furthermore, equipped with the tumor-targeting component HA, the co-delivery system shows specific targeting and high cellular uptake efficiency by receptor-mediated endocytosis. In summary, these dual-responsive (redox and pH), tumor-targeting smart 2-in-1 chimeric NPs show promise to be employed in functional co-delivery and tumor therapy.

## 1. Introduction

The co-delivery of small interfering RNA (siRNA) with chemotherapeutic drugs has attracted considerable attention because of improved anti-tumor efficacy over single-regimen administration [1,2]. In particular, co-delivering siRNA and chemotherapy drugs in a single nanocarrier proved more effective than sequentially delivering them in two separate nanocarriers [3]. However, the designs of drug co-delivery systems must be improved for better performance.

It is currently difficult to design unitary nanocarriers capable of encapsulating chemo drugs (e.g., hydrophobic small molecules) and siRNA (e.g., high-molecular-weight and polyanionic nature) because of the different physicochemical properties of each nanocarrier. Moreover, successful co-delivery of anti-cancer drugs and siRNA depends on the precise control of the timings at which the two agents are released in place [4]. The design of stimuli-responsive carriers can elevate physiological specificity and the on-demand therapeutic efficacy delivery of therapeutic agents in response to specific stimuli, either exogenous or endogenous [5]. In particular, redox-responsive properties also show increased potential because of the redox environment in the intracellular space. The concentration of the reducing agent glutathione (GSH) in the cytosol (approximately 2–10 mM) is much higher than that in the mildly oxidizing extracellular space, at approximately 2–20 μM. Additionally, the intracellular release of drugs can be responded to by the different concentrations of GSH in tumor tissues compared with healthy ones [4,6]. Furthermore, the slight differences in pH changes in tumor tissue (pH 6.5–6.8), endosomes (pH 5.5–6.5), lysosomes (pH 4.5–5.5) and blood and normal tissue (pH 7.4) have been extensively used to trigger the disassembly of pH-responsive delivery systems and cargo drugs into the cytosol via endolysosomal acidification and escape. Combination of oxidative environment and pH gradient in certain pathological conditions can promote the efficacy of drug delivery, resulting in an improvement of the therapeutic effect [7,8].

Chitosan (CS)-based polyelectrolyte complexes have received much attention in the field of drug delivery systems because they are formed by ionic interactions between two oppositely charged polyelectrolytes. Furthermore, their pH-stimuli-responsive capabilities can be used for controlled release purposes [9]. N,O-carboxymethyl chitosan (NOCC) is a derivative bearing a carboxymethyl polymer with amino and primary hydroxyl sites of the glucosamine units of the CS structure. NOCC is a more hydrophilic and amphoteric polyelectrolyte with a predominant cationic character suitable for biomedical applications [10,11]. Additionally, anionic hyaluronic acid (HA) spontaneously interacts with carboxymethyl chitosan, enhancing the drug-loading capacity and providing pH-responsive drug release behavior [12,13]. Furthermore, HA was used as a tumor-targeting ligand and enhances the internalization of various therapeutic agents through HA-binding receptors, such as the cell surface glycoprotein CD44 and receptor for HA-mediated motility.

Calcium phosphate (CaP) nanoparticles (NPs) are considered promising nucleic acid delivery carriers because of their biocompatibility and biodegradability and efficacious gene condensation. However, CaP encounters several critical drawbacks, limiting its clinical application, such as uncontrollable growth of crystals, lower reproducibility, and lack of tissue specificity [14,15,16]. Most strategies have been employed to stabilize CaP particles and deliver siRNA effectively based on the surface coating of CaP NPs, such as combining with PEGylated moieties [17,18], lipid bilayers [19] or polymers [20]. Among them, the catechol moiety has a strong affinity and enriches the organic–inorganic interface with calcium [21]. With the 3,4-Dihydroxy-L-phenylalanine (Dopa) structure, polymer enables efficient binding to the surface of CaP particles to inhibit further growth in a simple and green fabrication process [22,23].

**Scheme 1:**
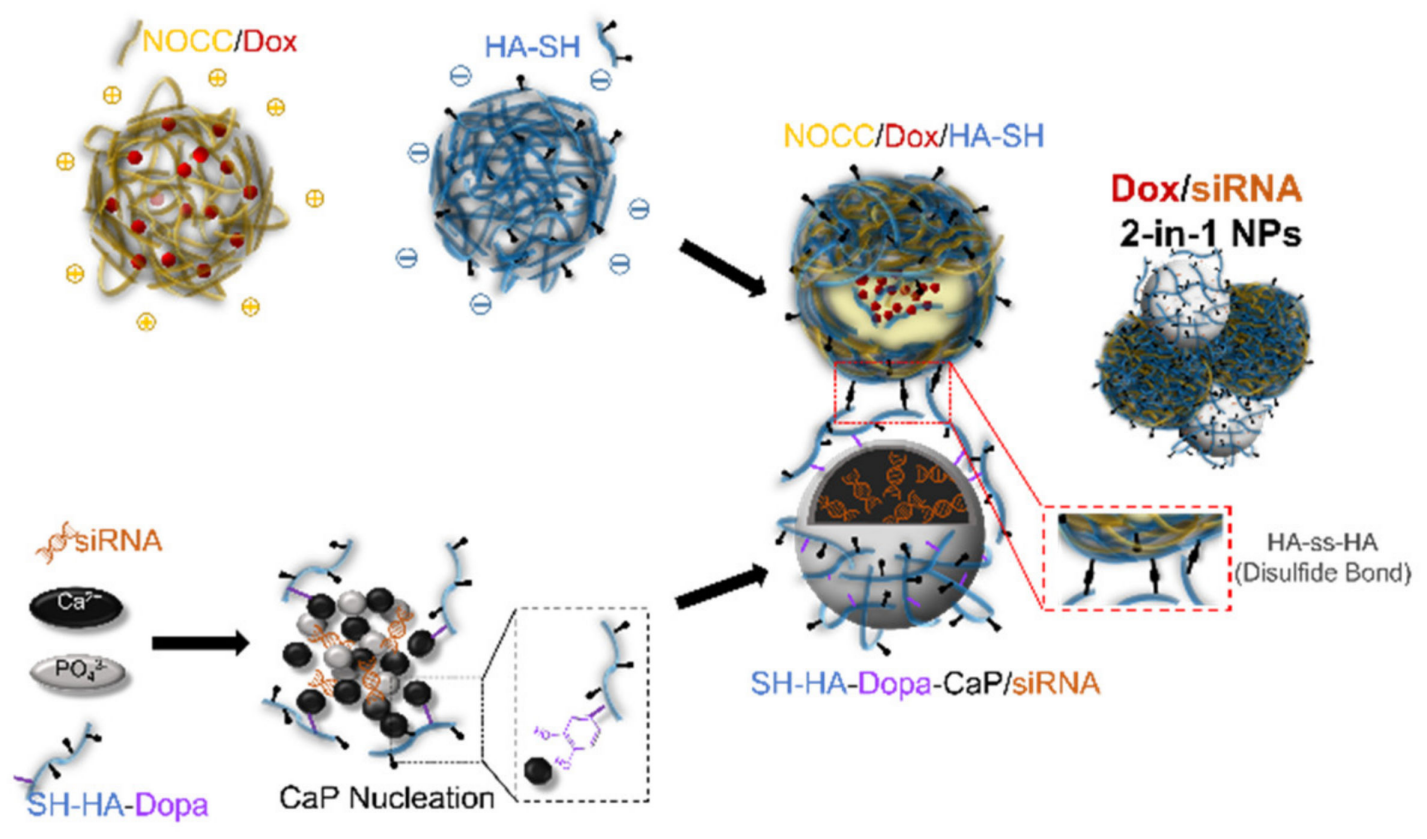
Schematic illustrations of the 2-in-1 chimeric delivery system constructed from individual Dox-loaded NOCC/HA-SH nanocarriers and SH-HA-Dopa-coated CaP/siRNA NPs conjugated through disulfide bond linkage (HA-ss-HA) to form an integrated vehicle.

Herein, we designed a targeted co-delivery therapeutic nanoagent constructed from two different components conjugated through a redox-sensitive thiol–disulfide bond to form one integrated nanovehicle (Scheme 1). Doxorubicin (Dox)-loaded NOCC complex with thiolated HA (HA-SH) forming spontaneously through ionic interaction as a pH-responsive polyionic nanocomplex for controlled release purposes. The electrostatic interactions between siRNA and CaP allowed siRNA to be condensed efficiently, and catechol-modified HA-SH acted like an outer shell to bind tightly and prevent further CaP crystal growth. In the intracellular reducible environment, the redox-triggered destruction of Dox/siRNA 2-in-1 chimeric NPs could be selectively released into the cytosol, and their cleavage led to the release of Dox and siRNA.

## 2. Materials and Methods

### 2.1. Materials

Chitosan (CS, 200–500 kDa) was purchased from Charming & Beauty Co., Ltd. (Taipei, Taiwan). Sodium hyaluronic acid (HA, 310 kDa) was purchased from Carelife Technology Corp. (Taipei, Taiwan). 2-Aminoethanethiol (cysteamine) and 1-ethyl-3-(-3-dimethylaminopropyl) carbodiimide hydrochloride (EDC) was purchased from Acros Organics (Geel, Belgium). Sodium hydroxide, monochloroacetic acid, disodium hydrogen phosphate heptahydrate, calcium chloride, isopropyl alcohol, dopamine hydrochloride (Dopa), *N*-hydroxysulfosuccinimide sodium (NHS), doxorubicin hydrochloride (Dox), HEPES, phosphate-buffered saline (PBS), glutathione (GSH) and Hoechst 33342 were purchased from Sigma-Aldrich (St. Louis, MO, USA). Negative control siRNA (siNC, sense strand: 5′-UUC UCC GAA CGU GUC ACG UTT-3′, anti-sense: 5′-ACG UGA CAC GUU CGG AGA ATT-3′), FAM-labeled negative control siRNA (FAM-siRNA) and Bcl-2 siRNA (siBcl-2, sense: 5′-GUA CAU CCA UUA UAA GCU GdTdT-3′, anti-sense: 5′-CAG CUU AUA AUG GAU GUA C-dTdT-3′) were purchased from GenePharma Co., Ltd. (Shanghai, China). Nuclease-free water and 5,5′-dithio-bis-(2-nitrobenzoic acid) (Ellman’s reagent) were purchased from Thermo Fisher Scientific (Waltham, MA, USA). A Qubit™ RNA BR assay kit was purchased from Invitrogen (Waltham, MA, USA). Cell Proliferation Reagent WST-1 was purchased from Roche (Basel, Switzerland).

### 2.2. Synthesis of N,O-Carboxymethyl Chitosan (NOCC), Thiolated Hyaluronic Acid (HA-SH) and Dopa-Conjugated Thiolated Hyaluronic Acid (SH-HA-Dopa)

NOCC was prepared according to Chen et al. with slight modifications [24]. CS powder was suspended in isopropyl alcohol and stirred at room temperature. Next, 25.2 mL of 10 N sodium hydroxide solution was divided into six equal parts and then was added to the stirred slurry. Monochloroacetic acid (12 g) was batch added to the reaction mixture for 3 h at 60 °C. Next, the pH value of the reaction solution was adjusted to 7. The solid was filtered and rinsed in 75% ethyl alcohol to desalt and dewater and dried using a freeze-drying method. HA-SH and SH-HA-Dopa were synthesized according to previous studies [4]. EDC (1 mmol) and NHS (1 mmol) were added to HA (0.125 mmol) in PBS buffer (pH 5.5) and then were reacted for 2 h. Cysteamine (1 mmol) and Dopa (0.5 mmol) were added to the previous mixture and stirred for 24 h. After the reaction, the product solution was purified by dialysis (MWCO = 3500 Da). The products were dried using the freeze-drying method and preserved at −20 °C before use. The chemical structures were characterized by ^1^H-NMR spectroscopy (AVANCE 500 NMR, Bruker, Germany). The thiol–disulfide exchange reaction was investigated using Ellman’s assay for the quantification of free sulfhydryl. The degree of disulfide bond formation was estimated by comparison to the free thiol of prepared sample in fresh.

### 2.3. Preparation and Characterization of Dox/siRNA 2-in-1 Chimeric NPs

To prepare NOCC/Dox/HA-SH NPs, 80 μL of NOCC solution (6 mg/mL) was added to 5 μL of Dox solution (1 mg/mL), followed by mixing with 2.5 μL of HA-SH solution (8 mg/mL). The mixed solutions were incubated at room temperature for 10 min to achieve equilibrium. To prepare SH-HA-Dopa-CaP/siRNA NPs, 30 μL of 500 mM CaCl_2_ solution (pH 7.4) was added to 30 μL of 10 μM siRNA solution for 1-min incubation. Subsequently, 60 μL of Ca^2+^/siRNA solution was quickly mixed with an equal volume of HEPES/phosphate/SH-HA-Dopa solution (50 mM HEPES, 280 mM NaCl, 1.5 mM Na_2_HPO_4_, 1 mg/mL SH-HA-Dopa, pH 7.4) with vortexing and allowed to react for 15 min. Finally, the Dox/siRNA 2-in-1 NPs were obtained after mixing with NOCC/Dox/HA-SH NPs and SH-HA-Dopa-CaP/siRNA NPs via disulfide bonds as intermediate linkers. The particle size, polydispersity index and zeta potential of the NPs were determined using a Nano-ZS90 zetasizer. The morphology of the NPs was observed by transmission electron microscopy (TEM; Hitachi H-7100). The Dox encapsulation efficiency was detected by UV-vis spectroscopy at 485 nm, and siRNA was detected using a Qubit™ RNA BR assay kit (Carlsbad, CA, USA). The encapsulated siRNA stability of NPs was mixed with fetal bovine serum (FBS) at the volume ratio of 1:1 and incubated at 37 °C for predetermined periods of time (0, 1, 3, 6, 12, and 24 h). The stability of the siRNA was analyzed by electrophoresis in a 2% agarose gel.

### 2.4. Drug Loading and Triggered Release

The freeze-dried delivery NPs were mixed with release media for incubation at 37 °C at different time intervals. PBS at different pH values (7.4, 6.5, and 5.0) with/without GSH was selected as the medium to mimic biological and early/late endosomal conditions. Later, the nanoparticle-containing solution was separated by centrifugation at 15,000 rpm for 10 min. The supernatant was collected and followed by quantitative analysis as described previously. Encapsulation efficiency (EE %) was calculated using below formula:

$$\mathrm{EE}\;\left(\%\right)=\frac{\left(C_{\mathrm{total}}-C_{\mathrm{free}}\right)}{C_{\mathrm{total}}}\times 100\%$$

where C_total_ was the concentration of total Dox or siRNA in the mixture and C_free_ represented the concentration of free Dox or siRNA unloaded or released by the NPs.

### 2.5. In Vitro Cytotoxicity

The WST-1 assay was performed to evaluate the in vitro cytotoxicity and therapeutic activity of NPs in HeLa cells. The cells were seeded in 96-well plates (8 × 10^3^ cells/well) and incubated for one night. The medium was replaced with medium containing NOCC/HA-SH NPs (0~3 mg/mL) and SH-HA-Dopa-CaP/siNC (siRNA: 0~800 nM). For the cytotoxicity of NPs, the concentrations of NOCC/HA-SH NPs and SH-HA-Dopa-CaP/siNC NPs were incubated for 72 h to determine cell viability. Samples containing only cells were set as the control group. After that, the cells were washed and incubated in fresh medium with 10% WST-1 reagent for another 4 h at 37 °C. Next, the optical density (OD) values at 450 nm were measured using a microplate reader. Cell viability (CV) was calculated as follows:

$$\mathrm{Cell}\;\mathrm{viability}\;\left(\%\right)=\frac{\left(\mathrm{Abs}._{\mathrm{sample}}-\mathrm{Abs}._{\mathrm{blank}}\right)}{\left(\mathrm{Abs}._{\mathrm{control}}-\mathrm{Abs}._{\mathrm{blank}}\right)}\times 100\%$$

where Abs._blank_, Abs._control_, and Abs._sample_ represent the OD values of the blank group (medium only), control group, and treatment group, respectively.

### 2.6. Cellular Uptake

The cellular uptake behaviors of the NPs in HeLa cell lines were analyzed using a fluorescence microscope (FM) based on Dox (10 µg/mL per well) and fluorescence-labeled FAM-siRNA (100 nM/well) from the individual delivery carrier on the NPs.

The cells were seeded (4 × 10^5^/well) onto 12-well culture plates and grown overnight. Cells were treated with free Dox, NOCC/Dox/HA-SH, naked FAM-labeled siRNA, SH-HA-Dopa-CaP/FAM-labeled siRNA or Dox/siRNA 2-in-1 NPs for incubation. After that, the cell nucleus was stained with Hoechst 33342, followed by observation with FM for internalization. The fluorescence intensity of Dox and FAM-labeled siRNA was measured separately using a microplate reader (excitation/emission wavelengths of 480/588 nm for Dox and 494/520 nm for FAM-labeled siRNA). The relative fluorescence intensity was calculated as follows:

$$\mathrm{Relative}\;\mathrm{fluorescence}\;\mathrm{intensity}\;\left(\%\right)=\frac{\mathrm{mean}\;\mathrm{fluorescence}\;\mathrm{intensity}\;\left(\mathrm{experimental}\right)}{\mathrm{mean}\;\mathrm{fluorescence}\;\mathrm{intensity}\;\left(\mathrm{control}\right)}\times 100\%$$

To study HA receptor (such as CD44) mediated cellular uptake of NPs in HeLa cells, an experiment was performed in the presence of free HA as competitor ligand. HA (10 mg/mL) was added to block the cell surface adhesion receptor, which promoted the uptake of NPs via receptor-mediated endocytosis for 30 min of pretreatment. The cellular uptake efficiency was determined as described above.

### 2.7. In Vitro Therapeutic Activity

The OVCAR-3 cell line was used to evaluate the in vitro therapeutic activity of the NPs using the WST-1 assay as described above. After refreshing the medium containing free Dox, NOCC/Dox/HA-SH, SH-HA-Dopa-CaP/siBcl-2, and Dox/siBcl-2 HNP (Dox: 5 μg/mL; siRNA: 50 nM), the cells were incubated for an additional 48 h. The FITC Annexin V Apoptosis Detection Kit I (BD Pharmingen™, San Diego, CA, USA) was carried out for quantitative analysis of cell apoptosis. OVCAR-3 cells were seeded in 6-well plates at a density of 5 × 10^5^ cells/well and cultured overnight, then incubated with different formulations. After 48 h exposure, cells were harvested and stained according to the manufacturer’s protocol. The stained cells were immediately assessed by the flow cytometer. Data analysis was performed using FlowJo software to quantify the numbers of cells undergoing necrosis and late apoptosis (both annexin V and PI positive), early apoptosis (annexin V positive and PI negative). Cells that are considered viable are both Annexin V and PI negative.

### 2.8. Statistical Analysis

All the experiments were evaluated in triplicate independently. Quantitative data were shown as means ± standard deviation. Statistically significant differences were assessed using Student’s *t*-test. Values of *p* < 0.05 were represented statistically significant.

## 3. Results

### 3.1. Synthesis and Characterization of NOCC, HA-SH and SH-HA-Dopa

**Figure 1:**
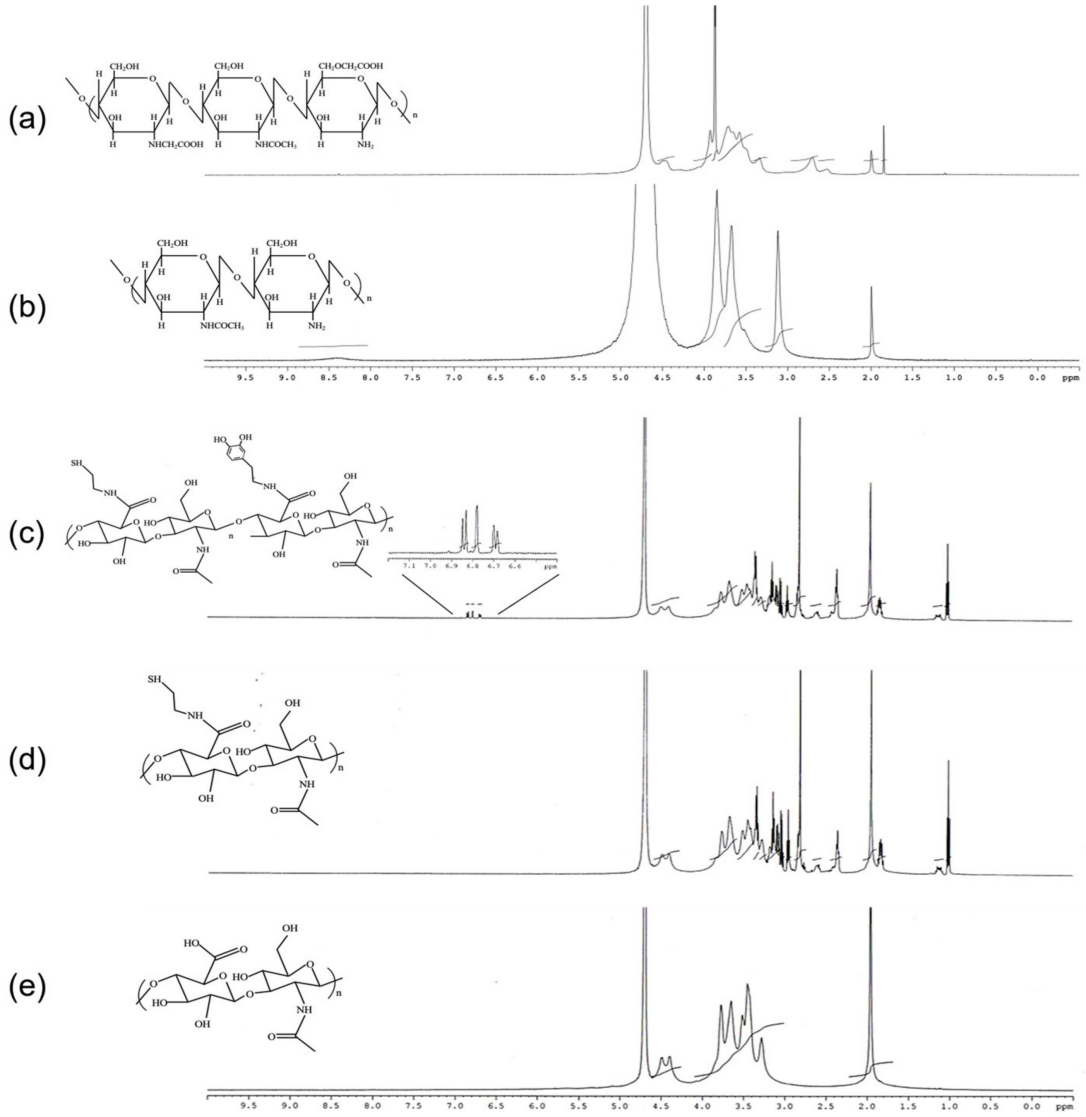
^1^H-NMR spectra of (**a**) NOCC, (**b**) CS, (**c**) SH-HA-Dopa, (**d**) HA-SH and (**e**) HA.

The chemical structures of NOCC, HA-SH and SH-HA-Dopa were evaluated by ^1^H-NMR. The chemical shifts of NOCC at 2.7 and 4.4 ppm were the protons of -N-CH_2_-COO and -O-CH_2_-COO at the N-position and O-position of the CS, respectively (Figure 1b) [25]. This finding showed that carboxymethyl substituents were observed on partial of the amino and primary hydroxyl sites of NOCC structure. However, both HA-SH and SH-HA-Dopa were prepared using an EDC/NHS coupling reaction. Cysteamine and Dopa were then grafted to HA by an amidation reaction. The structure of HA-SH was verified by the characteristic peaks between 2.5 ppm and 3.0 ppm (-NH-CH_2_-CH_2_), which represented the successful conjugation of thiol to HA (Figure 1d). Likewise, SH-HA-Dopa analysis demonstrated that the characteristic peaks of HA-SH were retained and that protons in the catechol ring were newly presented at 6.0–7.0 ppm [26]. The results showed that the coupling of Dopa to HA-SH was also achieved (Figure 1c). Next, we quantified the degree of substitution (DS) of the thiol group at HA-SH and SH-HA-Dopa, and the DS were 5.6 ± 0.2% and 4.9 ± 0.2%, respectively, using the Ellman’s assay.

### 3.2. Formation of NOCC/Dox/HA-SH, SH-HA-Dopa-CaP/siRNA and Dox/siRNA 2-in-1 Chimeric NPs

**Figure 2:**
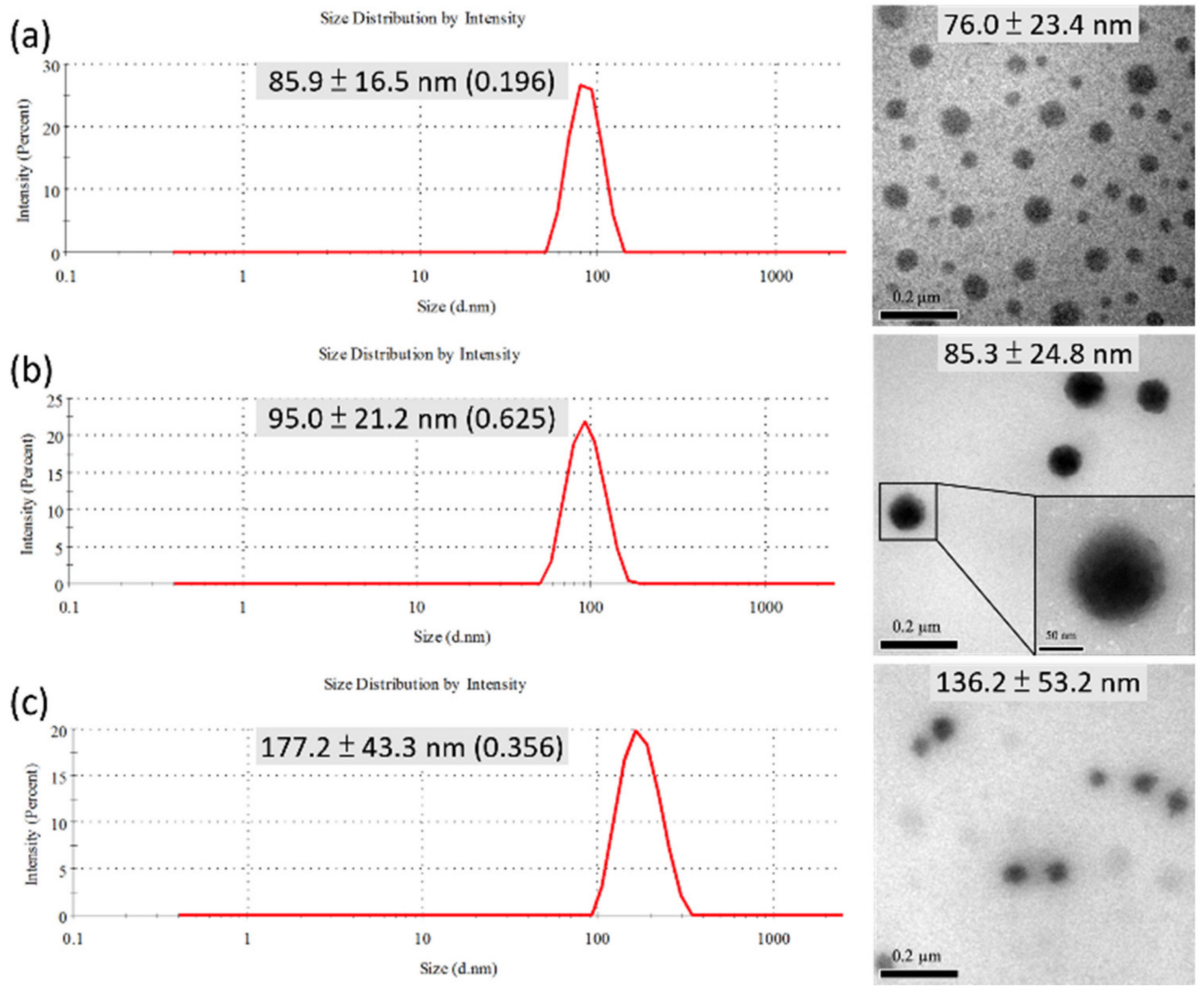
TEM images and DLS measurements (average particle size (PdI)) of (**a**) NOCC/Dox/HA-SH, (**b**) SH-HA-Dopa-CaP/siRNA and (**c**) Dox/siRNA 2-in-1 chimeric NPs.

In the present study, a 2-in-1 co-delivery system was constructed from a drug delivery nanocarrier (NOCC/Dox/HA-SH) and gene delivery NPs (SH-HA-Dopa-CaP/siRNA). All the groups had a nanoscale, good dispersion, and spherical morphology (Figure 2). From TEM images, the average size of NOCC/Dox/HA-SH, SH-HA-Dopa-CaP/siRNA and Dox/siRNA 2-in-1 chimeric NPs were approximately 76 nm, 85 nm and 136 nm, respectively. The DLS mean is approximately 11% higher than the TEM mean size. The slight change in the hydrodynamic size may be due to hydrodynamic diameter in addition to the contribution from the polymeric shell, which was hardly seen on the TEM images.

Individually, the average EE% values of Dox and siRNA were 73.3 ± 1.4% and 89.8 ± 0.1%, respectively. For the drug part of the co-delivery system, HA-SH was used as a polyanion to act as an ionic crosslinker through polyelectrolyte complexation with NOCC polycations and the formation of interfacial NOCC/HA NP complexes [27]. The model drug Dox was successfully encapsulated through physical adsorption and electrostatic interactions to form a NOCC/Dox/HA-SH nanocarrier (Scheme 1). For the siRNA part of the co-delivery system, the positive charge of calcium ions in CaP and the negative charge of the phosphate groups of nucleic acids revealed the binding affinity through electrostatic interactions (Scheme 1). A catechol group of Dopa provides efficient binding to metal ions such as Ca^2+^ in the CaP series [28]. The catechol moiety was chemically modified through EDC/NHS introduced into HA to improve the interactions between CaP-siRNA and SH-HA through the bioadhesive of Dopa to form the SH-HA-Dopa-CaP/siRNA transfection complex [29]. SH-HA-Dopa was anchored and observed around the surface of CaP-siRNA (Figure 2b). Additionally, the stability of SH-HA-Dopa-CaP/siRNA NPs could load siRNA and also provides effective protection to siRNA from serum degradation as shown in Supplementary Materials Figure S1. Therefore, SH-HA-Dopa not only effectively acts as a protective shell that prevents excessive growth and agglomeration of CaP NPs but also improved the thiolated HA interaction with CaP-siRNA to stabilize the gene carrier.

HA-SH, which served as a superior decoration on the surface of the carrier, was used to facilitate the formation of crosslinked HA-ss-HA in 2-in-1 chimeric NPs. The structure of 2-in-1 chimeric NPs builds collectively because of the crosslinking of the thiol groups and the building of disulfide bonds. The zeta potential measurements demonstrated that the surface charges of each carrier were negative (Table 1). In particular, the abundant surface charge of the carrier (−16~−30 mV) appeared to display the tendency to prevent aggregation of adjacent particles through electrostatic repulsion. Additionally, HA-SH was performed to facilitate the formation of disulfide bond oxidation linkages to form integrated 2-in-1 chimeric NPs. The size of our co-delivery system met the condition of enhanced permeability and retention (EPR), which would promote the accumulation of NPs in tumors [30].

### 3.3. In Vitro Cytotoxicity Studies

**Figure 3:**
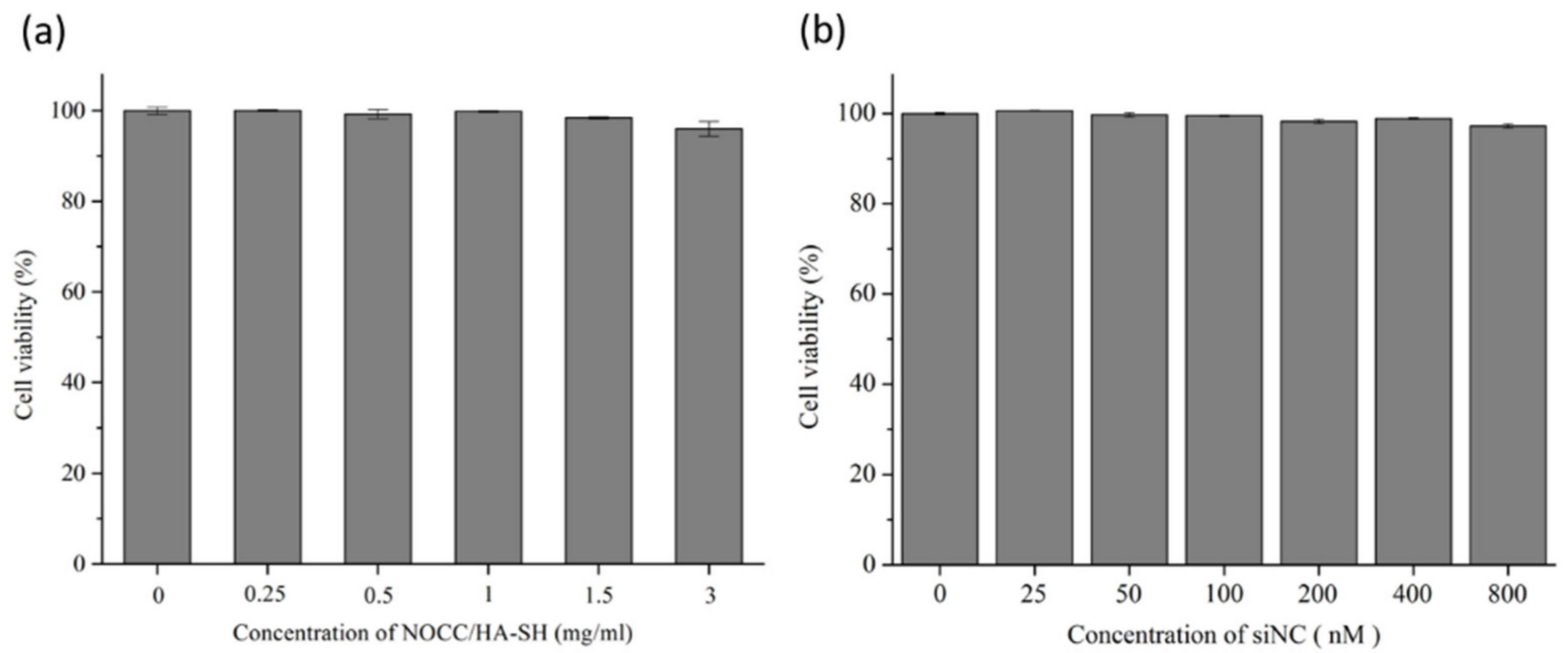
In vitro cytotoxicity of (**a**) NOCC/HA-SH and (**b**) SH-HA-Dopa-CaP/siNC to HeLa cells after 72-h incubation at 37 °C and 5% CO_2_. The data are shown as means ± SD (*n* = 3).

The WST-1 was performed to evaluate the cytotoxicity of NOCC/HA-SH and SH-HA-Dopa-CaP/siNC to HeLa cells individually. Compared with the blank control group, the treatment group under tested concentrations of NOCC/HA-SH and SH-HA-Dopa-CaP/siNC showed cell viability higher than 95% and revealed no significant distinction, indicating that the carriers have good biocompatibility at the tested concentration (Figure 3). Together with these observations, this developed co-delivery system may have limited cytotoxic effects when applied as a drug and siRNA carrier.

### 3.4. Release of Payloads in Response to pH and Redox Stimulation

**Figure 4:**
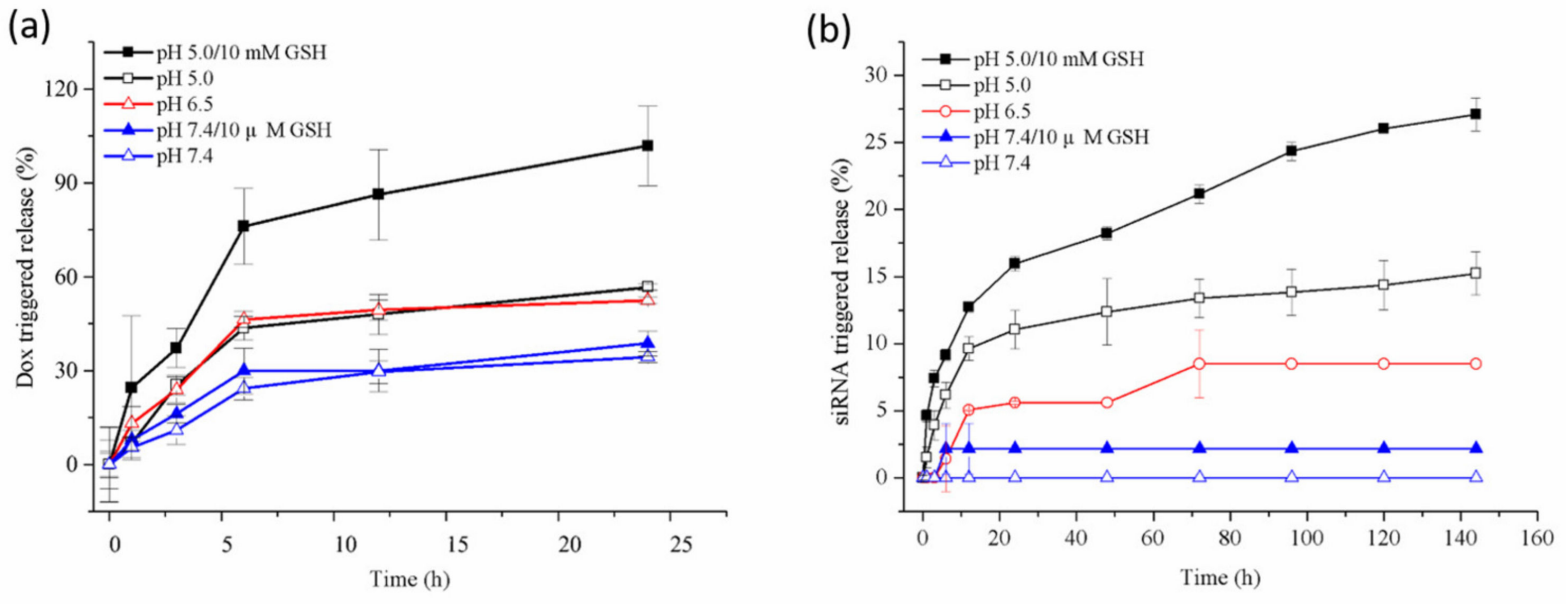
In vitro pH/redox stimuli release profiles from (**a**) NOCC/Dox/HA-SH and (**b**) SH-HA-Dopa-CaP/siRNA.

Variations exist in the pH and GSH between normal and tumor tissues, and intra- and extracellular compartments. Concerning these differences, the timing dependence of the cumulative Dox/siRNA release from delivery carriers was investigated under different pH (7.4, 6.5, 5.0) in the absence and presence GSH (10 μM, 10 mM) conditions [31,32]. The accumulated amount of Dox from the NOCC/HA-SH drug carrier was approximately 56.7% and 52.4% at pH 5.0 and 6.5 in 24 h, respectively (Figure 4a). NOCC/Dox/HA-SH released 34.3% of Dox at pH 7.4, demonstrating strong encapsulation and low leakage in the physiological environment. The acidic media likely weakens the interaction between NOCC and HA-SH. Because of the increasing protonation of NOCC as the pH declined, the release of Dox from NOCC/HA-SH was facilitated by electrostatic repulsion. Additionally, the electrostatic interaction between the carrier and drug was weakened, and the dismantling of Dox increased, benefiting drug release [33]. Therefore, the cumulative release ratio of Dox in tumor tissue (mildly acidic) was higher than that under physiological environments (pH 7.4). Furthermore, we investigated the Dox cumulative release behavior from NOCC/Dox/HA-SH to simulate intracellular release in tumor tissues at pH 5.0 in the presence of 10 mM GSH (mimicking cytosol). The accumulative release ratio was high over 95%, almost twice as high at pH 5.0 in the absence of GSH. Additionally, no obvious change (less than 5%) was observed when the NOCC/HA-SH carrier was treated with or without 10 μM GSH at pH 7.4.

The release of siRNA from SH-HA-Dopa-CaP under pH/redox stimulation was also investigated (Figure 4b). The release of siRNA from SH-HA-Dopa-CaP/siRNA was significantly augmented from 0% to 5.6% and further increased to 11% when the pH was decreased from 7.4 to 6.5 and further decreased to 5.5 over 24 h. Because of the pH response, decreasing the pH of the media increased the cumulative release ratio of siRNA. This finding reveals that almost all of siRNA stayed in the SH-HA-Dopa-CaP/siRNA, likely due to the CaP being dissolved slightly in the acidic condition, which is favorable for drug diffusion. Additionally, siRNA release from the SH-HA-Dopa-CaP carrier significantly affected the massive release phenome in the presence of GSH, particularly at pH 5.0.

The surface charge of all groups was confirmed by the zeta-potential measurement after they were dispersed into PBS at the medium of pH 5.0 with 10 mM GSH for 1 h, shown in Supplementary Materials Table S1. The zeta-potential of the NOCC/Dox/HA-SH, SH-HA-Dopa-CaP/siRNA and Dox/siRNA 2-in-1 chimeric NPs became more positive (21.7 ± 3.5, −7.3 ± 10.4 and 15.9 ± 5.4 mV) in comparison with the neutral condition due to the protonation of the amino group in NOCC and HA deionizes. Furthermore, the size distribution of NPs was studied with the same condition using DLS. The average hydrodynamic diameter of all groups became broader, and the diameters more greatly changed (as shown in Supplementary Materials Figure S2). The acidic media probably weakens the interaction between NOCC and HA-SH. The NOCC/Dox/HA-SH showed partial dissociation. Moreover, the disulfide bonds of HA-ss-HA were destroyed by high GSH concentration, and the structure of the NPs became less compact. Taking the above results into consideration, it could be summarized that the Dox/siRNA 2-in-1 chimeric NPs had excellent stability in the typical pH of physiological environments, while it dissociated under acidic conditions with a high concentration of GSH (pH 5.0 with 10 mM GSH). This feature could be used for the targeted delivery of anticancer drugs through the tumor microenvironment responsive controlled release.

Both NOCC/Dox/HA-SH and SH-HA-Dopa-CaP/siRNA displayed dual (pH- and redox-) triggered release characters. The cumulative release ratio of Dox and siRNA was obviously facilitated in the elevated GSH environments and mildly acidic conditions, such as tumor tissue (pH 6.0) or intracellular endo-/lysosomes (pH 5.0). Notably, the boosting release profile can be attributed to the following characteristics of the carrier structure: decrease in the ionic crosslinking degree by elevating the protonation of NOCC and HA-SH; cleavage of the disulfide bonds in HA-ss-HA; and dissolution of the CaP. Instead, the carriers were relatively stable under normal physiological conditions. This feature of the 2-in-1 chimeric nanocarrier could be used for targeted delivery through controlled tumor microenvironment-responsive release.

### 3.5. Cellular Uptake, Internalization and Distribution

**Figure 5:**
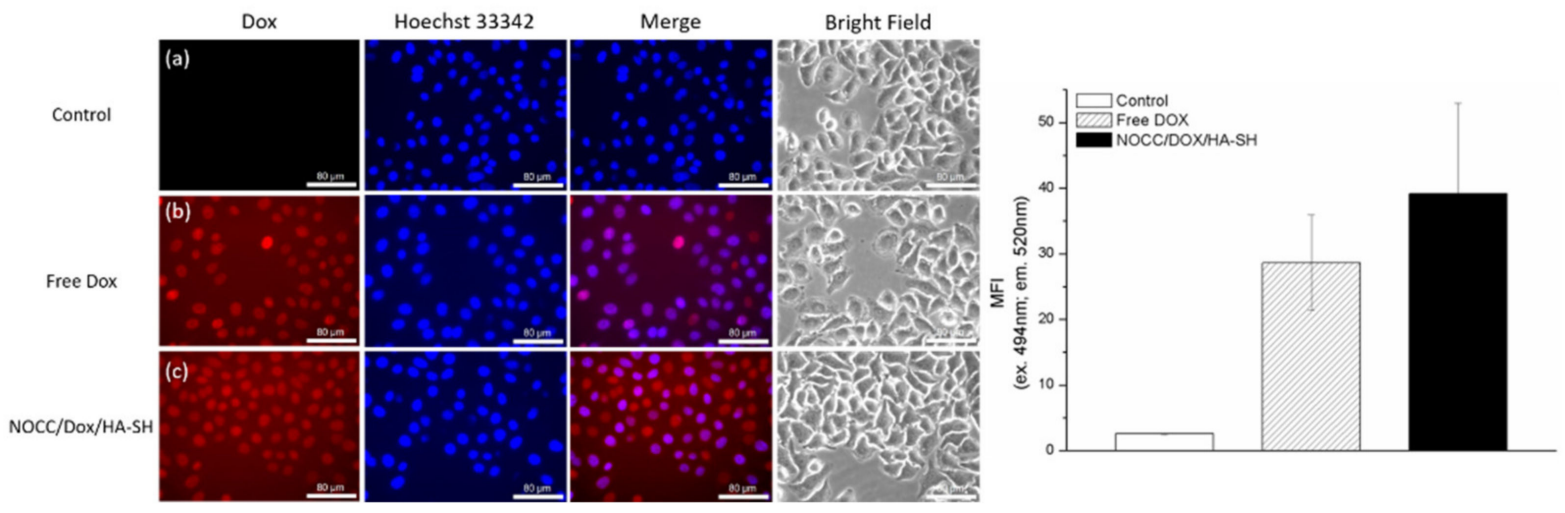
Cellular uptake of (**a**) blank, (**b**) free Dox, and (**c**) NOCC/Dox/HA-SH by fluorescence microscopy in HeLa cells after 8 h of incubation. The data are shown as means ± SD (n = 3).

**Figure 6:**
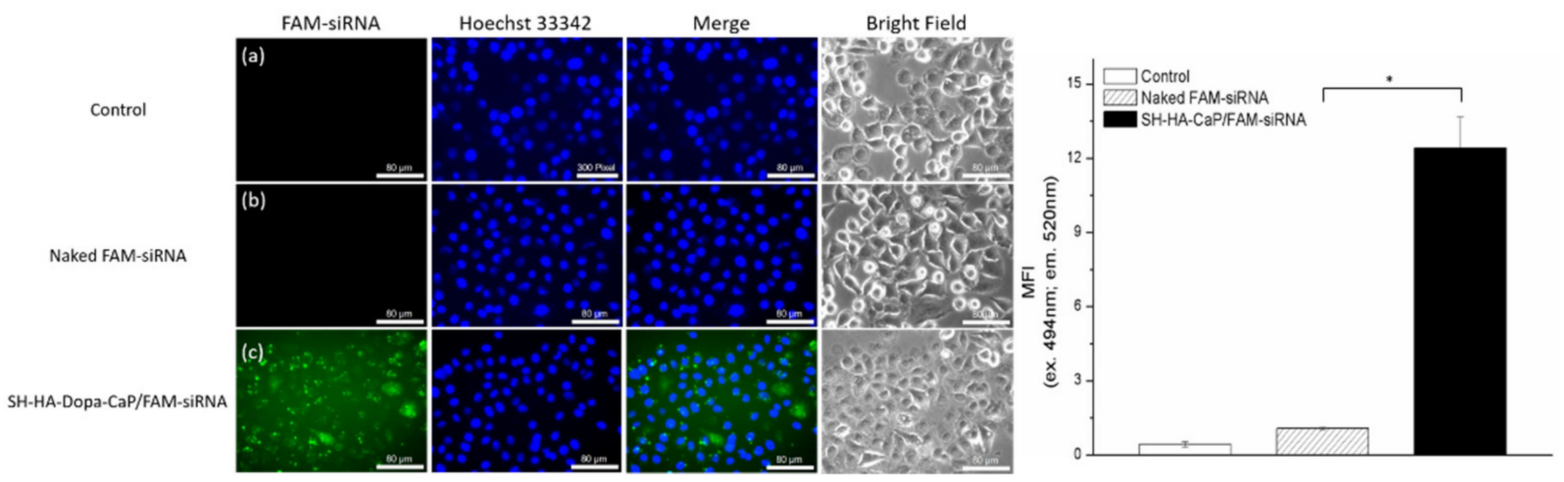
Cellular uptake of (**a**) blank, (**b**) FAM-siRNA, and (**c**) SH-HA-Dopa-CaP/FAM-siRNA (100 nM siRNA/well) after 2 h of incubation with HeLa cells. The data are shown as means ± SD (n = 3); * *p* < 0.01.

The effective delivery of therapeutic drugs remains a major hurdle, and ideal carriers must overcome many natural barriers to transport their cargo to the site of action. Fluorescence microscopy was used to observe the cellular uptake of NOCC/Dox/HA-SH and SH-HA-Dopa-CaP/siRNA nanocarriers. Dox shows red fluorescence, the nuclei were stained with Hoechst 33342 (blue), and green fluorescent dye (FAM)-labeled scrambled siRNA was replaced to locate the intracellular distribution of siRNA. Dox was more efficiently delivered into cells by NOCC/Dox/HA-SH than free drug (Figure 5). Additionally, FAM-siRNA was observed within the cytoplasm of HeLa cells, indicating the internalization of the SH-HA-Dopa-CaP/FAM-siRNA NPs (Figure 6c). The cells exhibited significant quenching of the fluorescent signal with FAM-siRNA alone, while cells treated with SH-HA-Dopa-CaP/FAM-siRNA presented much more (10 times) fluorescence (Figure 6b). After 2 h, FAM-siRNA exhibited an indiscernible fluorescent signal in the cytoplasm. However, by contrast, much stronger fluorescence was released from SH-HA-Dopa-CaP/FAM-siRNA NPs and filled whole cells. The SH-HA-Dopa-CaP delivery carrier not only facilitated cellular uptake of siRNA but also provided protection against nuclease attack. These results consistently show that NOCC/Dox/HA-SH and SH-HA-Dopa-CaP/siRNA effectively deliver Dox and siRNA into cells, respectively.

**Figure 7:**
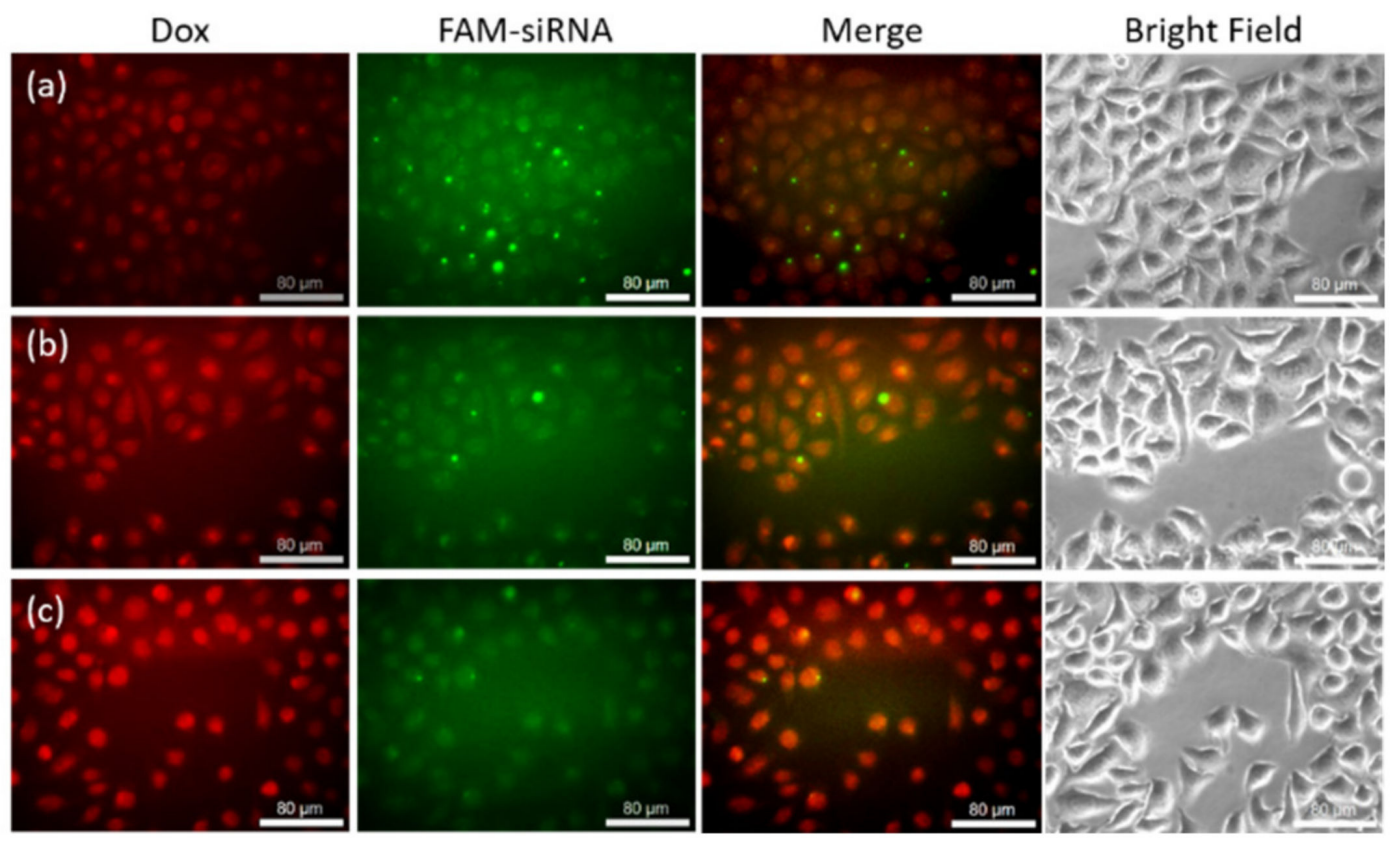
Cellular uptake behavior of Dox/FAM-siRNA chimeric NPs in HeLa cells after incubation for (**a**) 2, (**b**) 4, and (**c**) 8 h. Fluorescence images of cultures showing Dox (red), FAM-siRNA (green), merged images, and bright field images.

To achieve the best synergistic outcome of the combined siRNA and anti-cancer drugs, the payloads must not only be delivered in a single carrier simultaneously but also must be unloaded in a cancer cell at two segregate, precisely controlled timings. The intracellular delivery of Dox/FAM-siRNA 2-in-1 chimeric NPs was further evaluated. The cellular distributions of FAM-siRNA (green) and Dox (red) were both time-dependent. Dox and FAM-siRNA were both observed mainly in the cytoplasm during the initial 2 h (Figure 7a). The overlap of the two fluorescence-matched wells appeared yellow, indicating the effective co-delivery of the two therapeutic agents simultaneously. After incubation for 4 h, the orange-yellow co-localization signal decreased, indicating that Dox and FAM-siRNA were separated gradually with the incubation time (Figure 7b). The disulfide bridges in Dox/FAM-siRNA 2-in-1 chimeric NPs were cleaved in response to the intracellular reductive condition. Additionally, acidic pH as an internal stimulus has arisen as a trigger for the selective release of therapeutic drugs. Red and green fluorescence signals were again observed some distance apart, demonstrating the successful escape of Dox in NOCC/HA-SH and siRNA in SH-HA-Dopa-CaP from the lysosomes. These results can be contributed to the increased capability of the delivery system to release from lysosomes. After 8 h of treatment with Dox/FAM-siRNA chimeric NPs, more Dox was almost completely accumulated and migrated into nuclei, indicating that Dox was released from NOCC/Dox/HA-SH efficaciously. By comparison, the FAM-siRNA fluorescence was at a relatively low level in nuclei instead of within the cytoplasm (Figure 7c). The diffuse movement of Dox and siRNA from the endolysosomes into the cytoplasm were observed by the distribution of green and red signals and the approaching red regions to the nucleus.

**Figure 8:**
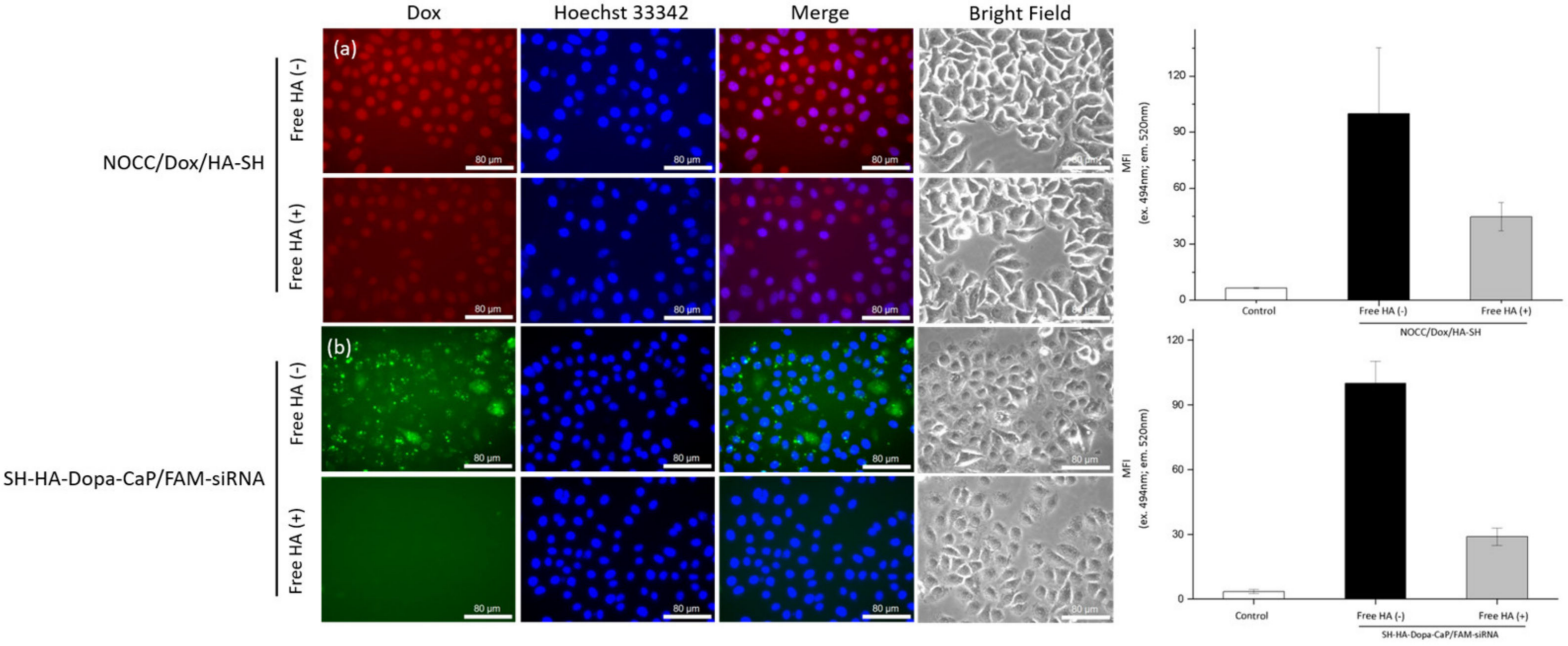
In vitro intracellular uptake of (**a**) NOCC/Dox/HA-SH and (**b**) SH-HA-Dopa-CaP/FAM-siRNA NPs after 30 min in the absence and presence of free HA (10 mg/mL).

The drug delivery efficiency is significantly enhanced by promoting the internalization of nanocarriers in dividing and non-dividing cells through receptor-mediated endocytosis [34]. The receptor-mediated cellular uptake of chimeric NPs was further investigated by determining the influence of free HA as a competitor ligand. The cellular uptake efficiency of either NOCC/Dox/HA-SH or SH-HA-Dopa-CaP/FAM-siRNA was significantly reduced by the presence of free HA (Figure 8). This result indicated enhanced specific cellular uptake of nanocarriers whose surface was decorated with HA derivatives. The superior endocytosis of therapeutic drugs can be explained by the increased cellular uptake via HA receptor-mediated endocytosis. The developed chimeric NPs are considered to deliver drugs directly into HA receptor overexpression tumoral tissue for active targeting.

### 3.6. Antitumor Effect

**Figure 9:**
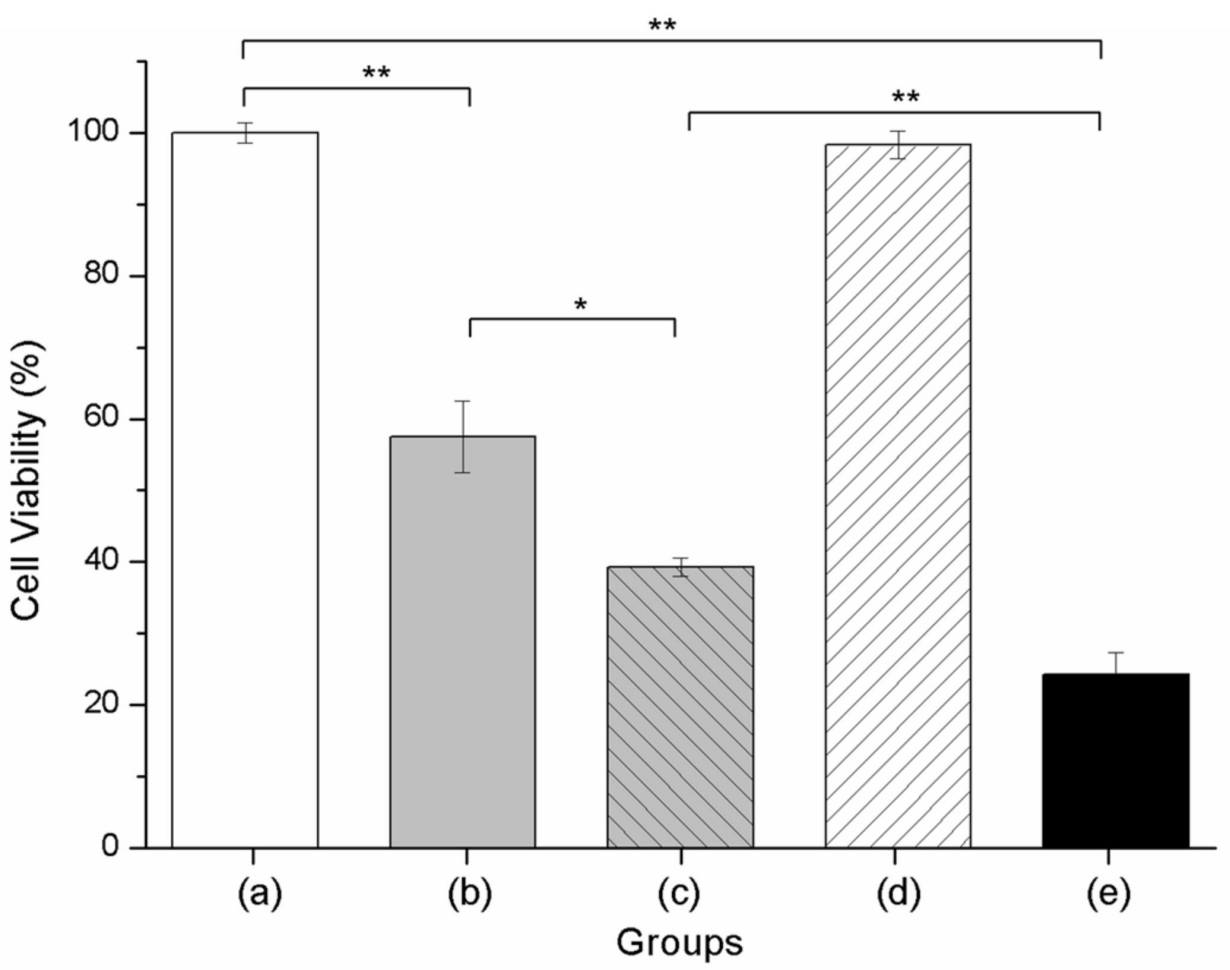
In vitro therapeutic activity of OVCAR-3 cells treated with different formulations: (**a**) control (cell only), (**b**) free Dox, (**c**) NOCC/Dox/HA-SH, (**d**) SH-HA-Dopa-CaP/siBcl-2, and (**e**) Dox/siBcl-2 chimeric NPs for 48 h of co-incubation. Data are show as mean ± SD (n = 3). * *p* < 0.01, ** *p* < 0.001.

The advantage of co-delivering anticancer drugs and genes using nanocarriers has shown promising results for overcoming multidrug resistance (MDR) in cancer cells. Anti-apoptotic Bcl-2 siRNA provides effective gene silencing and then restores the chemosensitivity of MDR cells [35]. The significant improvement of the chemotherapeutic effects of Dox/siBcl-2 2-in-1 chimeric NPs was investigated in OVCAR-3/MDR cells. The cell viability was significantly reduced by 43% after incubation with free Dox compared with the cell-only control group (Figure 9b). NOCC/Dox/HA-SH showed further enhanced intracellular uptake and resulted in an 18% reduction in cell survival compared with the same amount of free Dox (Figure 9c). NOCC/Dox/HA-SH could more efficiently enter OVCAR-3 cells via non-specific phagocytosis than free Dox through a passive diffusion mechanism. By comparison, Dox/siBcl-2 chimeric NP treatment further enhanced these antiproliferation effects, resulting in only 24.3% cell viability. However, the cytotoxicity of the SH-HA-Dopa-CaP/siBcl-2 group revealed no significant difference (Figure 9d). These results indicate that SH-HA-Dopa-CaP/siBcl-2 does not cause any severe cytotoxic effect and can be administrated for siRNA delivery in the appropriate concentration ranges to suppress the drug resistance-associated genes without undesirable cytotoxicity [36]. This result demonstrated that siBcl-2 could enhance the sensitivity of OVCAR-3/MDR cells to Dox treatment. This synergistic therapeutic effect was achieved when Dox and Bcl-2 siRNA were co-delivered by chimeric NPs (Figure 9e).

**Figure 10:**
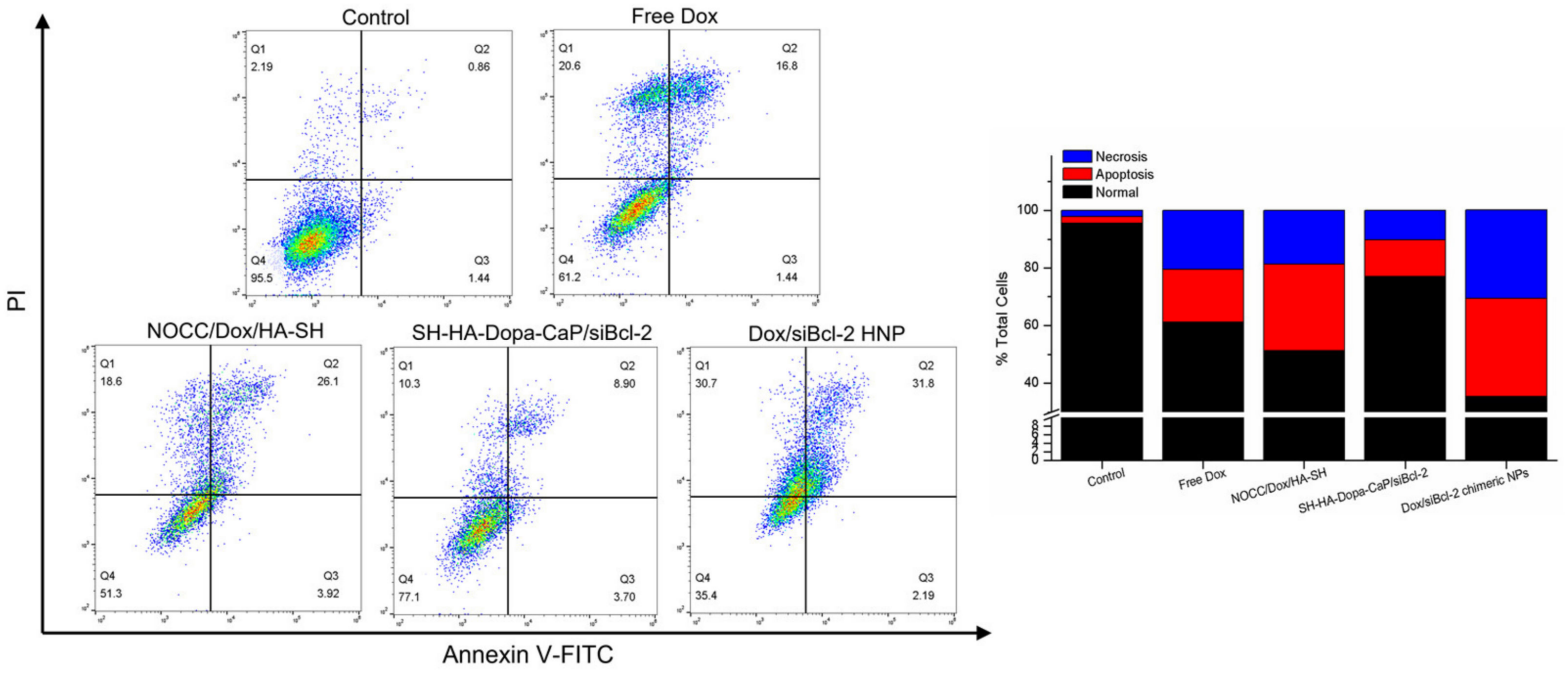
Flow cytometry analysis of OVCAR-3 cell apoptosis after different treatments using the Annexin V-FITC/PI assay and statistical analysis of normal, apoptotic, and necrotic cells. Data are shown as mean ± SD (n = 3).

Furthermore, the effect of Dox and Bcl-2 siRNA was quantitatively investigated by flow cytometry to examine whether the co-delivered Dox and Bcl-2 siRNA could induce cell apoptosis effectively (Figure 10). The percentage of apoptotic/necrotic cells in the Dox/siBcl-2 chimeric NPs group (64.6%) was much higher than those in the NOCC/Dox/HA-SH (48.6%), free Dox (38.8%) and SH-HA-Dopa-CaP/siBcl-2 (22.9%) groups. These results showed that treatment with the coexistence of therapeutic drug reveals the ideal antitumor effect, while the co-delivery system demonstrated a synergistic effect of diversified therapies to maximize the treatment effect of cancer.

## 4. Conclusions

This developed chemodrug/siRNA co-delivery system was constructed from individual NOCC/Dox/HA-SH (drug) and SH-HA-Dopa-CaP/siRNA (siRNA) conjugated through thiol–disulfide bonds to form one integrated nanovehicle. This innovative drug-delivery system is synthesized via a moderate ionic and covalent double-crosslinking method. Redox-sensitive 2-in-1 chimeric NPs are expected to have superior stability in the extracellular environment and provide rapid tumoral selective separation and release. These 2-in-1 chimeric NPs show specific targeting and effectively deliver the therapeutic cargo to cancer cells through HA receptor-mediated endocytosis. When Dox/siRNA was simultaneously delivered to tumor cells, apparent accumulation was observed, and the encapsulated drugs release was triggered by acidic pH values and high intracellular GSH concentration, resulting in improved antitumor efficacy. Furthermore, the 2-in-1 chimeric NPs provide therapeutic effectiveness superior to that of unity drug-loaded NPs. To sum up, the developed 2-in-1 chimeric NPs show promising co-delivery of Dox and siRNA nanocarriers with favorable cytocompatibility, tumor targetability, and accurate controlled release in specific microenvironmental changes that could be tailored for biological applications in cancer therapy.

## Figures and Tables

**Table 1:** Zeta potential of the NPs.

Sample	Zeta Potential (mV)
NOCC/Dox/HA-SH	−30.8 ± 4.19
SH-HA-Dopa-CaP/siRNA	−16.1 ± 1.71
Dox/siRNA 2-in-1 NPs	−20.2 ± 6.02

## Supplementary Materials

The following are available online at https://www.mdpi.com/article/10.3390/polym13244362/s1, Figure S1: Serum stability of (a) naked siRNA, (b) CaP/siRNA and (c) SH-HA-Dopa-CaP/siRNA, Figure S2: The hydrodynamic diameter distributions of (a) NOCC/Dox/HA-SH, (b) SH-HA-Dopa-CaP/siRNA and (c) Dox/siRNA 2-in-1 chimeric NPs at the medium of pH 5.0 with 10 mM GSH by DLS measurements, Table S1: Zeta potential of the NPs.
