# Supplementary material for: Redox/pH-Responsive 2-in-1 Chimeric Nanoparticles for the Co-Delivery of Doxorubicin and siRNA
Source: Polymers (Basel). 2021 Dec 13;13(24):4362. doi: 10.3390/polym13244362 (PMC8703840; doi:10.3390/polym13244362)
Supplement: Supplementary file 1 [file polymers-13-04362-s001.zip › polymers-1429554-supplementary.pdf]

Supplementary Material

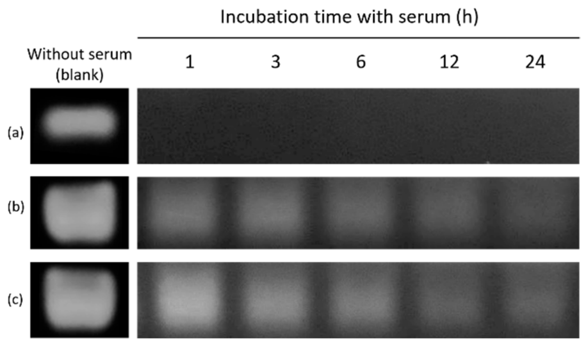

Figure S1. Serum stability of (a) naked siRNA, (b) CaP/siRNA and (c) SH-HA-Dopa-CaP/siRNA.

Table S1. Zeta potential of the NPs.

Sample	Zeta potential (mV)
NOCC/Dox/HA-SH	21.7 ± 3.5
SH-HA-Dopa-CaP/siRNA	-7.3 ± 10.4
Dox/siRNA HNP	15.9 ± 5.4

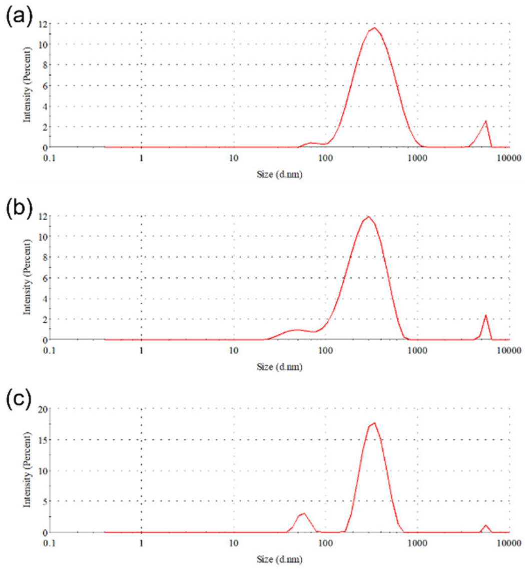

Figure S2. The hydrodynamic diameter distributions of (a) NOCC/Dox/HA-SH, (b) SH-HA-Dopa-CaP/siRNA and (c) Dox/siRNA 2-in-1 chimeric NPs at the medium of pH 5.0 with 10 mM GSH by DLS measurements.
